# Gestational Diabetes Mellitus Worsens the Profile of Cardiometabolic Risk Markers and Decrease Indexes of Beta-Cell Function Independently of Insulin Resistance in Nondiabetic Women with a Parental History of Type 2 Diabetes

**DOI:** 10.1155/2014/743495

**Published:** 2014-07-06

**Authors:** Alina Sokup, Barbara Ruszkowska-Ciastek, Małgorzata Walentowicz-Sadłecka, Marek Grabiec, Danuta Rość

**Affiliations:** ^1^Department of Gastroenterology, Angiology and Internal Diseases, Nicolaus Copernicus University, Dr. J. Biziel University Hospital, Ujejskiego 75, 85-168 Bydgoszcz, Poland; ^2^Department of Endocrinology, Dr. J. Biziel University Hospital, Ujejskiego 75, 85-168 Bydgoszcz, Poland; ^3^Department of Pathophysiology, Nicolaus Copernicus University, Dr. A. Jurasz University Hospital, Skłodowskiej-Curie 9, 85-094 Bydgoszcz, Poland; ^4^Department of Obstetrics and Gynecology, Nicolaus Copernicus University, Dr. J. Biziel University Hospital, Ujejskiego 75, 85-168 Bydgoszcz, Poland

## Abstract

*Background*. Women with a history of both parental type 2 diabetes (pt2DM) and previous gestational diabetes (pGDM) represent a group at high risk of cardiovascular events. We hypothesized that pGDM changes cardiometabolic risk markers levels as well as theirs associations with glucose indices in nondiabetic pt2DM women. *Methods*. Anthropometric parameters, glucose regulation (OGTT), insulin resistance (HOMA-IR), beta-cell function, lipid levels, parameters of endothelial dysfunction, and inflammation were evaluated in 55 women with pt2DM, 40 with both pt2DM and pGDM 2–24 months postpartum, and 35 controls. *Results*. Prediabetes was diagnosed more frequently in women with both pt2DM and pGDM in comparison with women with only pt2DM (10 versus 8, *P* = 0.04). The pGDM group had higher LDL-cholesterol, sICAM-1, tPa Ag, fibrinogen, and lower beta-cell function after adjustment for HOMA-IR, in comparison with pt2DM group. In pt2DM group postchallenge glucose correlated independently with hsCRP and in pGDM group fasting glucose with HOMA-IR. *Conclusions*. pGDM exerts a combined effect on cardiometabolic risk markers in women with pt2DM. In these women higher LDL-cholesterol, fibrinogen, sICAM-1, tPa Ag levels and decreased beta cell function are associated with pGDM independently of HOMA-IR index value. Fasting glucose is an important cardiometabolic risk marker and is independently associated with HOMA-IR.

## 1. Introduction

Prevention of cardiovascular morbidity and mortality has become major health issues worldwide. Previous history of gestational diabetes mellitus (pGDM) is an equivalent of a pathophysiologic state, which predisposes to the development of both diabetes and cardiovascular disease in young and adult women [1, 2]. In women with pGDM and additional parental history of type 2 diabetes (pt2DM), the risk of cardiovascular disease is higher and occurs at a younger age, in comparison to women with only confirmed pt2DM [1]. Interestingly, the increase in the frequency of cardiovascular disease events in this specific subpopulation of women with both pt2DM and pGDM is independent of metabolic syndrome and type 2 diabetes [1].

Up to date it is unknown was is the pathophysiologic conditioning of higher cardiovascular risk in women with pt2DM and observed post-GDM in comparison to pt2DM women but without pGDM. Taking into consideration the stable increase in the incidence of gestational diabetes mellitus (GDM) as well as type 2 diabetes worldwide, research into the cardiometabolic risk markers in this specific group of women with both pt2DM and pGDM should be especially important.

Numerous longitudinal studies indicate that there is relationship between fasting glucose [3], postprandial glucose concentrations [3], hemoglobin A1C [4], and cardiovascular risk markers in nondiabetic subjects. Additionally, cardiovascular risk extends into the nondiabetic range and is somewhat stronger for postprandial, rather than fasting glucose concentrations [3]. Similar results were obtained in studies including women with GDM during and after an index pregnancy [5, 6]. It has been reported that fasting glucose and 2 h postchallenge glucose assessed during oral glucose tolerance test at diagnosis of GDM [6], as well as after an index pregnancy [5], are associated with an increase of cardiometabolic risk in the future. In addition, impaired fasting glucose and impaired glucose tolerance assessed after pregnancy in these women exhibited different associations with traditional cardiovascular risk markers [5].

Thus, evaluation of different cardiometabolic risk markers with their relations to glucose indices may give more information about the pathophysiology of higher cardiometabolic risk in pGDM women than evaluation of cardiometabolic risk markers only.

So far there have been no studies aimed at assessing the high spectrum of traditional and nontraditional cardiometabolic risk parameters as well as its associations with glucose indices in nondiabetic Caucasian women with pt2DM after an index pregnancy complicated by GDM.

Our hypothesis was that cardiometabolic risk markers profile and/or pathophysiologic association between these markers and glucose indices would be more proatherogenic in nondiabetic women with both pt2DM and a previous history of GDM compared to women with only pt2DM. Thus results of our study could be useful in elaboration of individualized preventive strategies for women with both pt2DM and pGDM.

Therefore, the aim of the present investigation was to study whether the levels of cardiometabolic risk markers, beta-cell function, or insulin resistance degree differ in nondiabetic pt2DM women with a pGDM and in nondiabetic women with only pt2DM as well as in comparison with each of these two groups with control women without both pGDM and a pt2DM. Additionally we looked for the associations between glucose indices (fasting glucose, 2-hour postchallenge glucose, and A1C) and cardiometabolic risk markers in women with pt2dM and in women with both pt2DM and a pGDM.

## 2. Materials and Methods

This research was conducted in the Diabetes and Pregnancy Unit at the Outpatient Clinic of the Department of Endocrinology at the Dr. J. Biziel University Hospital in Bydgoszcz between 2002 and 2007. The studied population included 130 nondiabetic Caucasian women with a history of last pregnancy from 2 to 36 months. 55 women had a positive parental history of diabetes type 2 (pt2DM) and had a history of one or more pregnancies but had not a previous history of GDM (pGDM). The mean time of observation in this group was 14 months. 40 women with pt2DM who were diagnosed and treated at our department were evaluated within 2 to 24 months after GDM (mean 10 months). 35 healthy women who had one or more pregnancies, with neither history of pt2DM nor history of pGDM, presented control group, mean time of observation 18 months. To assess the glucose status during pregnancy in women who were entered into this study and were not treated at our department, we asked them about diagnostic procedures during pregnancy and about the presence or absence of GDM. Mean parity in these groups of women was 1,94 + −0,96 in pt2DM group, 2,10 + −1,20 in group with both pt2DM and pGDM, and 1,7 = −1,10, respectively, in control group. Most of pGDM women 26(65%) were breastfeeding during the first year postpartum. In group of women with only pt2dM 10(10%) and 7(20%) women in the control group were breastfeeding during evaluation. None of the participants had renal, hepatic, or cardiovascular disease. None of them were diagnosed with diabetes, nor were they taking medications affecting lipid or carbohydrate metabolism. Women with infections, as well as smokers, were excluded from the study. Informed written consent was obtained from all participants and the protocol was approved by the Local Research Ethics Committee of the University.

### 2.1. Methods of Diagnosis of GDM

Testing for GDM was performed in accordance with recommendations of the Polish Diabetes Society. All the pregnant women with no previously diagnosed diabetes were offered screening for gestational diabetes mellitus (GDM) using 1 h, 50 g oral glucose challenge test (GCT) between 24 and 28 weeks of pregnancy. If fasting plasma glucose evaluated at early prenatal visit was equal to or above 5.5 mmol/L (threshold ≥ 100 mg/dL), the GCT test was performed by the gynecologist as soon as possible. A value equal to or above 7.7 mmol/dL (threshold ≥ 140 mg/dL) identified women who underwent a further 2 h 75-g oral glucose tolerance test (OGTT). Diagnosis of GDM was in accordance with the World Health Organization criteria [7] adapted by the Polish Diabetes Society. GDM was diagnosed when the fasting plasma glucose was equal to or above 5.5 mmol/L (threshold ≥ 100 mg/dL) in two distinct tests, or when the plasma glucose was equal to or above 7.7 mmol/L (threshold ≥ 140 mg/dL) two hours after a 75 g oral glucose challenge test.

### 2.2. Study Methods

All participants underwent a physical examination that included anthropometric measurements. Waist circumference was measured at the smallest circumference between the ribs and the iliac crest. Body mass index (BMI) was calculated as weight in kilograms divided by the squared height in metres. The current status of glucose regulation was assessed using OGTT according to WHO procedure and defined according to the modified the World Health Organization recommendations [7] with the threshold of fasting normoglycaemia at 5.5 mmol/L (<100 mg/dL). Blood samples were obtained after 12-hour overnight fasting, and venous blood was drawn from the antecubital vein. Lipid profile was studied by measuring total cholesterol, low-density lipoprotein- (LDL-) cholesterol, high-density lipoprotein (HDL-) cholesterol, and triglycerides. Endothelial dysfunction was assessed by measuring soluble intracellular adhesion molecule-1(sICAM-1), soluble vascular cell adhesion molecule-1(sVCAM-1), soluble E-selectin (s-Es), tissue plasminogen activator antigen (tPa Ag), and von Willebrand factor antigen (vWFAg). Systemic inflammation was assessed by measuring plasminogen activator inhibitor-1 antigen (PAI-1Ag), high-sensitivity C-reactive protein (hsCRP), fibrinogen, and white blood cell count.

The severity of insulin resistance was studied by homeostatic method [8] using homeostasis model assessment-insulin resistance (HOMA-IR) index. Pancreatic beta-cell function was studied using homeostasis model assessment-beta-cell (HOMA-B) index, insulinogenic index, and insulinogenic index-to-HOMA-IR index ratio as disposition index.

### 2.3. Laboratory Assessment

The concentration of fibrinogen was measured by chronometric and chromogenic methods in an automated coagulometer CC-3003 apparatus, using reagents produced by Bio-Ksel Co, Poland. The number of WBC was determined by the hematology analyzer ADVIA 120, Siemens, Germany. Measurement of white blood cells was preceded by a cytochemical reaction, resulting in preparation of white blood cells in order for them to change color, allowing for their easy distinguishing.

The methods of the laboratory assessment of the other cardiovascular risk markers have been presented elsewhere [9].

## 3. Statistical Methods

Patient characteristics were described as mean ± SD, median, and interquartile range,* n*(%), and proportions. The analysed parameters were tested for normal distribution using the Shapiro-Wilk test. The variables were not normally distributed; the Kruskal-Wallis test was used with post hoc analysis to locate the differences. The Chi squared test was used to compare proportions. To exclude potential confounders we used regression analysis. Correlation coefficients were calculated and multivariate regression analysis was performed in order to determine associations between fasting glucose, glucose at 2 h ater 75 g glucose loading, HbA1C, and the values of assessed cardiovascular risk markers.* P* values of <0.05 were considered statistically significant. The statistical analysis was carried out using Statistica 8.0 (StatSoft, Cracow, Poland).

## 4. Results

### 4.1. Baseline Clinical Characteristics

The mean age of 130 women was 29.9 years, and their mean BMI was relatively low 23.3 kg/m^2^. The prevalence of prediabetic state in the whole population was 38 (29%).

In women with both pt2DM and pGDM, the subgroup with prediabetes consisted of 4/40(10%) women with normal fasting glucose and impaired glucose tolerance (NFG/IGT), 5/40(12%) women with impaired fasting glucose and normal glucose tolerance (IFG/NGT), and 1/40(3%) woman with impaired fasting glucose and impaired glucose tolerance (IFG/IGT). In the pt2DM group, subgroup of prediabetes consisted of 4/55(7%) women with NFG/IGT, 4/55(7%) women with IFG/NGT. In all subjects of the control group the results of OGTT were normal.

### 4.2. Cardiometabolic Characteristics of the pt2DM/pGDM Women, pt2DM Women, and Control Women with No History of pt2DM and pGDM

All women were stratified into three groups: women with both pt2DM and pGDM, women with pt2DM only, and controls.

There were no differences between these groups in terms of age, fasting glucose, 2 h postchallenge glucose, HbA1C, BMI, waist circumference, and other assessed parameters.

Women with pt2DM versus controls exhibited higher insulin resistance index HOMA-IR, higher soluble E-Selectin (s-Es), and higher triglycerides-to-HDL-cholesterol ratio after adjustment for BMI, 2 h postchallenge glucose, and waist circumference. Women with pt2DM and a history of pGDM versus controls exhibited lower insulinogenic index, lower disposition index, higher concentration of total cholesterol, LDL-cholesterol, total-to-HDL-cholesterol ratio, triglycerides-to-HDL-cholesterol ratio, fibrinogen, sICAM-1, s-Es, tPa Ag concentrations, and higher leukocyte count after adjustment for BMI and 2 h postchallenge glucose. The difference between women with both pt2DM and pGDM and women without pGDM was higher LDL-cholesterol, sICAM-1, tPa Ag, fibrinogen concentrations, lower insulinogenic index, and disposition index after adjustment for HOMA-IR. HOMA-IR index was lower in women with pGDM. Prediabetes occurs more frequently in women after GDM in comparison with women with only pt2DM. There was significant difference between the numbers of women with prediabetes in both groups. The results of statistical analysis are shown in Table 1.

### 4.3. Associations between Cardiometabolic Risk Markers and Fasting Glucose, 2-Hour Postchallenge Glucose, and Haemoglobin A1C in Women pt2DM /pGDM and pt2DM

In the second analysis we looked for an association between fasting glucose, 2 h postchallenge glucose, hemoglobin A1C (HbA1C), and cardiometabolic risk parameters using correlation analysis and multivariate regression analysis.

In the group of pt2DM women fasting glucose correlated positively with sICAM-1 level (*r* = 0.38), 2 h postchallenge glucose with LDL-cholesterol-to-HDL-cholesterol ratio (*r* = 0.27), insulin (*r* = 0.33), hs-CRP (*r* = 0.37), and HOMA-IR (*r* = 0.31). Using multivariate regression analysis 2 h glucose was independently associated with hs-CRP only. A1C was positively correlated with triglycerides-to-HDL-cholesterol ratio, total-to-HDL-cholesterol ratio and LDL-to-HDL-cholesterol ratio (*r* = 0.30), total-cholesterol-to-HDL-cholesterol (*r* = 0.34), and LDL-cholesterol-to-HDL-cholesterol ratio (*r* = 0.29). There were no independent associations between HbA1C and analysed variables.

In the pGDM group fasting glucose was positively correlated with BMI (*r* = 0.38), waist circumference (*r* = 0.42), triglycerides-to-HDL-cholesterol ratio (*r* = 0.56), insulin concentration (*r* = 0.71), and HOMA-IR (*r* = 0.76). In multivariate regression analysis fasting glucose was independently associated only with HOMA-IR. Results of multivariate analysis are shown in Tables 2, 3, and 4.

## 5. Discussion

The present study was designed to examine the possible pathophysiologic mechanisms which may explain the increase of cardiometabolic risk in nondiabetic women with parental history of type 2 diabetes which occurs after gestational diabetes mellitus (GDM). In this study we show several interesting findings potentially associated with this problem. We report that nondiabetic women with parental history of type 2 diabetes observed post-GDM differ in comparison with women with only pt2DM with respect to the cardiometabolic risk; (1) prediabetes occurs more frequently in women with pt2DM and with pGDM; (2) beta-cell function is characterized by the decrease of early phase of insulin hypersecretion, decrease of disposition index as well as decrease of insulin resistance degree to the normal range; (3) cardiometabolic risk profile is more proatherogenic independently of insulin resistance index; (4) glucose indices, such as fasting plasma glucose and 2 h postchallenge glucose and haemoglobin A1C, show different patterns of associations with cardiometabolic risk factors which may suggest changes of associations between cardiometabolic risk factors and pathophysiologic mechanisms of glucose regulation.

Interestingly, almost two times higher prevalence of prediabetes in women observed after GDM than in women with parental diabetes with pt2DM but without GDM is also higher than the case of other populations of women assessed after GDM [10]. We know that different types of glucose dysregulation defined as prediabetes are associated with a higher risk of both diabetes and cardiovascular disease in general population. Thus, this observation indicates the necessity of prevention of diabetes in women with a parental diabetes type 2 with a pGDM more rigorously than in women with only parental type 2 diabetes.

Other interesting observations in our study are the decrease of beta-cell function and decrease of index HOMA of insulin resistance to the normal range in women with parental history of type 2 diabetes assessed after GDM. These pathophysiologic changes, especially decrease of disposition index, may increase the risk of development of glucose dysregulation and diabetes in women with parental diabetes type 2 in their future life [11]. Our results are in agreement with another study, where post-GDM status was independently associated with diminished first phase of insulin secretion assessed during intravenous glucose tolerance test, as well as decreased insulinogenic index even after adjustment for intrahepatocellular fat [12]. It is plausible that the alterations in beta cell function, reduced early insulin secretion, and decreased disposition index observed in our pt2DM women after GDM can be attributed to the specific detrimental influence of GDM, which is similar to those observed in other women studied after GDM [13]. Our results are in disagreement with the results of another study, which points at hepatic insulin resistance as a cause of early decline in beta-cell function postpartum in women with glucose intolerance during pregnancy [14]. These discrepancies may point at differences in pathophysiologic mechanisms of beta-cell dysfunction in studied populations of women after GDM.

In our current study we show for the first time proatherogenic changes of cardiovascular risk profile in women with parental history of type 2 diabetes which we observed after GDM. In these women cardiovascular risk markers indicate higher systemic inflammation, higher LDL-cholesterol level, and higher endothelial dysfunction independently of insulin resistance index HOMA-IR. It is noteworthy also that this index was in normal range; for example, it was similar to control group. From the other hand it is well known that there is association between HOMA-IR and cardiovascular risk markers in women after GDM even at the normal range of this index which is characteristic feature of post-GDM state.

Thus, our finding suggests that higher levels of fibrinogen, LDL-cholesterol, sICAM-1, and tPa Ag have not pathophysiologic relations to hepatic insulin resistance index, a characteristic feature of post-GDM state [11, 12]; however, higher levels of these parameters partly explain higher rate of cardiovascular events in women with parental diabetes type 2 observed after pGDM compared to women with only pt2DM [1]. So, the increasing of hepatic insulin sensitivity will not be effective in reducing these cardiovascular risk factors in women with parental diabetes in prevention strategies proposed in these women after GDM [11]. Taken together, the profile of cardiometabolic risk in women with pt2DM with a history of GDM assessed by plasma concentrations of circulating markers and beta-cell indexes is similar to profile observed in other populations of women with pGDM [15–18] but is not dependent on mainly hepatic insulin resistance index HOMA-IR. Because it is known that breastfeeding decreases cardiometabolic risk in post-GDM women by different mechanisms [19]; thus, it may partly influence most of our results, for example, insulin resistance, beta-cell function, and cardiovascular risk markers levels in group evaluated after GDM where most of women were breastfeeding during examination.

Taken into consideration that endothelial dysfunction in normoglycemic first degree relatives of type 2 diabetic patients is independent of metabolic syndrome [20, 21] and that pGDM is associated with endothelial dysfunction (higher s-Es and sVCAM-1 concentrations) after an index pregnancy [22], results of our current study may suggest rather an additive than interactive character of pathophysiologic mechanisms due to the family history of type 2 diabetes and pGDM. Similar pathophysiologic mechanisms may explain higher concentrations of fibrinogen [15], LDL cholesterol [17, 18], and tPa Ag [16] in women with parental diabetes type 2 observed after GDM in our study.

As noted earlier, there are data suggesting that glucose indices such as fasting plasma glucose, 2-hour postchallenge glucose, and haemoglobin A1C assessed during pregnancy complicated by GDM and postpartum are associated with cardiometabolic risk [5, 6]. Our study performed in nondiabetic women with parental type 2 diabetes with and without previous GDM postpregnancy revealed interesting pathophysiologic results. In pt2DM women fasting glucose, 2 h postchallenge glucose and A1C are associated with cardiometabolic risk markers and the level of hs-CRP seems to be an important marker and a tool in preventive strategies aimed at postchallenge glucose in these women. Post-GDM fasting glucose seems to be a marker of cardiometabolic risk, which is a consequence of primarily basal insulin resistance (HOMA-IR) per se at similar to control group value of HOMA-IR index. Thus, improving hepatic insulin sensitivity probably due to reduced hepatic steatosis [11] should be important in preventive strategies. Reducing of hepatic insulin resistance should be advantageous in reduction of cardiometabolic risk associated with higher surrogate measures of insulin resistance with lower triglyceride-to-HDL-cholesterol ratio strictly associated with the ratio of small and dense particles of LDL-cholesterol lipoprotein. This finding is in agreement with results of a previous study, in which in pGDM women a stronger relationship was observed between impaired fasting glycaemia than impaired glucose tolerance and obesity and hypertension [5]. Our current findings are in disagreement with suggestions that insulin resistance [23, 24], especially hepatic insulin resistance, is the key pathophysiologic mechanism associated with cardiovascular risk markers in pGDM women. These discrepancies suggest pathophysiological differences between pGDM women with and without pt2DM.

Finally, the latter findings may suggest that GDM changes not only the cardiometabolic risk profile, but also the relationships between its markers with pathophysiological factors affecting fasting glycemia, 2 h postchallenge glucose, and glucose dysregulation represented by A1C. This suggestion needs to be further examined and may be important for preventive strategies in pt2dM women with as well as without pGDM.

A possible limitation of our study is the use of fasting state HOMA as a means of estimating insulin resistance. The HOMA-insulin resistance index is widely used to evaluate insulin resistance and is well correlated with the results of clamp studies. Additionally, results of some studies point at the pathophysiological significance of hepatic insulin resistance [16, 25], rather than the whole-body insulin resistance in pGDM women.

## 6. Conclusions

A previous history of gestational diabetes mellitus is associated with several pathophysiologic changes, which occur in nondiabetic women with parental type 2 diabetes and may partly explain higher cardiometabolic risk in their future life. Results of our study indicate a higher risk of diabetes, more proatherogenic cardiometabolic risk profile with decreased pancreatic beta-cell function independently of value of insulin resistance index HOMA-IR which decreases to the normal range after an index pregnancy. Association of fasting plasma glucose concentration with surrogate measures of insulin resistance with independent association of fasting glucose with HOMA-IR indicates that the fasting glucose is an important cardiometabolic risk marker and insulin resistance index HOMA-IR even in the normal range is important target in preventive management in pt2DM women with a previous GDM. Thus, our current findings suggest some differences in preventive strategy in women with parental type 2 diabetes and in women with parental type 2 diabetes withn abadditional history of GDM.

## Figures and Tables

**Table 1:** Clinical characteristics of the study participants stratified into three groups: women with both pt2DM and pGDM, women with pt2DM but without pGDM, and control group.

Variable	pt2DM + pGDM *N* = 40 (I)	pt2DM *N* = 55 (II)	Controls *N* = 35 (III)	*P*
Age (years)	31.63 (4.73)	29.18 (5.89)	29.25 (5.32)	0.0622
BMI (kg/m^2^)	25.25 (4.44)	25.23 (5.15)	22.08 (2.06)	0.0020^I–III^ 0.0069^II-III^
Waist (cm)	83.61 (14.43)	82.93 (17.23)	74.03 (6.91)	0.0475^II-III^
Fasting glucose (mmol/L)	4.88 (0.62)	4.78 (0.66)	4.63 (0.53)	0.3740
2 h (OGTT) glucose (mmol/L)	6.27 (1.65)	5.63 (1.50)	4.97 (0.60)	0.0001^I–IIIb^ 0.0411^II-III^
HbA1C (%)	5.65 (5.20–5.90)	5.50 (5.20–5.80)	5.40 (5.30–5.40)	0.0858
Fasting insulin (pmol/L)	51.00 (36.00–80.40)	72.72 (59.28–97.44)	54.60 (47.40–61.80)	0.0808^I-IIa^ 0.3034^II-IIId^
HOMA R	1.67 (1.29–2.29)	2.67 (2.13–3.42)	1.89 (1.58–2.37)	0.0039^I-II^ 0.0015^II-III^
HOMA B	130.04 (97.79–187.23)	209.90 (135.17–282.53)	159.00 (119.44–186.12)	0.35^I-IIa^
Insulinogenic index	16.38 (9.55–27.54)	36.49 (19.76–70.09)	27.45 (14.44–36.79)	<0.0001^I-IIa^ <0.0001^I–IIIbc^
Disposition index	8.79 (5.01–14.81)	12.92 (6.90–26.52)	16.70 (7.22–17.48)	0.0497^I-IIa^ 0.0498^I–IIIbc^
Total cholesterol (mmol/L)	5.07 (4.50–5.74)	4.80 (4.28–5.61)	4.56 (4.27–5.04)	0.0162^I–IIIbc^
LDL-cholesterol (mmol/L)	3.21 (2.72–3.75)	2.61 (2.20–3.08)	2.57 (2.30–3.05)	0.0006^I-IIa^ 0.0010^I–IIIbc^
HDL cholesterol (mmol/L)	1.55 (1.27–1.86)	1.55 (1.27–1.82)	1.73 (1.56–1.82)	0.0717^I–IIIbc^ 0.0705^II-III^
Triglycerides (mmol/L)	1.12 (0.72–1.53)	1.06 (0.69–1.50)	0.86 (0.67–1.05)	0.0800^I–IIIbc^ 0.1072^II-III^
TG/HDL-cholesterol	1.56 (1.00–2.63)	1.38 (0.97–2.77)	1.11 (0.89–1.50)	0.0347^II-IIId^
Total./HDL cholesterol	3.42 (2.71–4.17)	2.96 (2.50–3.89)	2.70 (2.37–3.18)	0.0029^I–IIIbc^
LDL-/HDL cholesterol	2.11 (1.57–2.76)	1.64 (1.25–2.23)	1.56 (1.18–1.89)	0.070^I-IIa^ 0.14^I–IIIbc^
Hs-CRP (ng/mL)	1.60 (0.70–3.28)	0.55 (0.27–2.97)	0.41 (0.22–0.79)	0.10^I–IIIbc^
Leukocyte count	6720.00 (6060.00–7500.00)	6100.00 (5100.00–7600.00)	5290.00 (4800.00–6540.00)	0.0032^I–IIIbc^
Fibrinogen	2.80 (2.50–3.30)	2.30 (1.90–2.60)	2.13 (1.90–3.00) 0.12	0.0003^I-IIa^ 0.0054^I–IIIbc^
PAI-1Ag (ng/mL)	56.59 (38.70–88.62)	67.20 (42.15–101.01)	71.36 (50.54–81.80)	0.4390
sVCAM-1 (ng/mL)	1105.43 (466.68–1509.53)	678.30 (565.85–981.90)	672.05 (455.70–1113.46)	0.1314
sICAM-1 (ng/mL)	305.01 (254.12–365.07)	167.02 (129.79–209.80)	140.40 (121.34–179.90)	<0.0001^I-IIa^ <0.0001^I–IIIbc^
sSel E (ng/mL)	35.18 (25.73–41.68)	30.34 (17.31–47.37)	20.90 (17.11–26.01)	0.0018^I–IIIbc^ 0.0351^II-IIId^
vWFAg (ng/mL)	104.45 (94.90–114.34)	97.08 (83.80–114.73)	104.87 (74.96–125.67)	0.3250
t-PaAg (ng/mL)	4.77 (3.81–7.13)	3.32 (2.20–5.70)	3.50 (2.66–4.64)	0.0147^I-IIa^ 0.0221^I–IIIbc^
Prediabetes	10 (15%)	8 (14%)		0.04∗

All data are shown as mean (± SD), medians (IQR-interquartile range), *P* values refer for differences between the three groups by using Kuskall-Wallis test. ^a^Adjusted for HOMA IR, ^b^adjusted for BMI, ^c^adjusted for postchallenge 2 h glucose, ^d^adjusted for BMI, waist circumference and postchallenge 2 h glucose, and ∗hi squared test.

**Table 2:** Multivariate regression analysis for dependent variable glucose at 2 h OGTT in women with pt2DM.

Variables	Beta	*B*	*P*
LDL cholesterol/HDL cholesterol	0.15	0.27	0.36
HOMA-IR	0.11	0.10	0.50
hs-CRP	0.32	0.13	0.04

*R* ^2^ = 0.17.

**Table 3:** Multivariable regression analysis for dependent variable HbA1C in women with pt2DM.

Variables	Beta	*B*	*P*
Triglycerides/HDL cholesterol	−0.46	−0.67	0.40
Total Cholesterol/HDL cholesterol	2.67	0.81	0.07
LDL-Cholesterol/HDL cholesterol	−1.88	−0.80	0.10
Triglycerides/HDL cholesterol	0.10	−0.56	0.57
Delta glucose at 30′ OGTT	0.26	0.05	0.22
HOMA-IR	−0.20	0.04	0.29
BMI	−0.35	−0.98	0.33
Waist circumference	0.33	0.01	0.34

*R* ^2^ = 0.39.

**Table 4:** Multivariate regression analysis for dependent variable fasting glucose in women with both pt2dM and a history of GDM.

Variables	Beta	*B*	*P*
BMI	0.15	−0.25	0.51
Waist circumference	0.06	−0.03	0.82
Triglycerides/HDL cholesterol	0.10	−0.56	0.57
HOMA-IR	0.77	−3.82	0.0008

*R* ^2^ = 0.53.

## Abbreviations

GDM: Gestational diabetes mellitus
pGDM: Previous history of gestational diabetes mellitus
pt2DM: Parental history of type 2 diabetes mellitus
OGTT: Oral glucose tolerance test
A1C: Glycated haemoglobin A1C
ICAM-1: Intercellular adhesion molecule-1
VCAM-1: Vascular cell adhesion molecule-1
tPa Ag: Tissue plasminogen activator antigen
PAI-1 Ag: Plasminogen activator inhibitor antigen
hs-CRP: High sensitivity C-reactive protein
s-ES: Soluble E-selectin
NFG/IGT: Normal fasting glucose and impaired glucose tolerance
IFG/NGT: Impaired fasting glucose and normal glucose tolerance
IFG/IGT: Impaired fasting glucose and impaired glucose tolerance.
